# Preventative management against thromboembolism using fresh frozen plasma in a coronary artery bypass graft patient with protein S deficiency: a case report

**DOI:** 10.1186/s40981-018-0155-4

**Published:** 2018-02-15

**Authors:** Kenzaburou Sugimoto, Mamoru Kadosaki, Atsushi Egawa, Rina Tokitou, Miho Urayama, Mamoru Takeuchi

**Affiliations:** 1 Department of Anesthesiology and Critical Care Medicine, Jichi Medical University, 3311-1, Yakushiji, Shimotsuke-City, Tochigi 329-0498 Japan; 2 Department of Pediatric Intensive Care and Anesthesia, Jichi Children’s Medical Center Tochigi, 3311-1, Yakushiji, Shimotsuke-City, Tochigi 329-0498 Japan

**Keywords:** Protein S deficiency, Coronary artery bypass grafting, Fresh frozen plasma, Thromboembolism, Cardiopulmonary bypass

## Abstract

Protein S deficiency patient is characterized by recurrent thrombosis, and its risk is higher intraoperatively, especially in cardiac surgery involving cardiopulmonary bypass. Two heparin cessation periods are defined in cardiac surgery. One is the period between the cessation of heparin 4 to 5 h before surgery and the heparinization prior to cardiopulmonary bypass. The other is the period between protamine administration and resumption of heparin postoperatively. Because the risk of thromboembolism is high during the period of heparin cessation, other anticoagulants are necessary. Although fresh frozen plasma, rich in protein S, is often used in cardiac surgery for protein S deficiency patients, the most appropriate times and volume of its administration to prevent thromboembolism remain poorly understood. We herein report a case of on-pump coronary artery bypass grafting in a patient with protein S deficiency who received fresh frozen plasma targeting the two heparin cessation periods. Some qualitative measurements to identify the effect of fresh frozen plasma on the protein S level are desirable to evaluate whether our present administration strategy has any beneficial effects on protein S deficiency patients.

## Background

Protein S (PS) serves as a cofactor for protein C (PC), an inhibitor of activated coagulation factors V and VIII, resulting in anticoagulation [1]. PS deficiency is characterized by recurrent thrombosis, thus anticoagulants such as heparin or warfarin are strongly recommended perioperatively [2]. However, heparin must be discontinued intraoperatively and the risk of thromboembolism is high during the period of heparin cessation, especially in patients undergoing cardiac surgery involving cardiopulmonary bypass (CPB).

Although fresh frozen plasma (FFP) is rich in PS and is often used in cardiac surgery for patients with PS deficiency [3, 4], the most appropriate times and volume of its administration to prevent thromboembolism remain to be clarified.

## Case presentation

A 58-year-old man (height, 160 cm; weight, 64 kg) presented with exertional dyspnea 6 months ago. Cardiac catheterization showed a 90% stenosis at each branch of left and right coronary arteries. The patient responded to medical therapy; thus, elective on-pump coronary artery bypass grafting (CABG) was scheduled.

He had a history of deep vein thrombosis and multiple cerebral infarctions at 46 years of age. Screening for a thrombophilic diathesis was performed at that time and showed a reduced PS activity level of 33.7% (reference range, 70–160%), while the PC activity level was normal at 123% (reference range, 70–130%). The significant difference between the PS and PC activity levels strongly suggested PS deficiency. He had no comorbidities to which acquired PS deficiency could be attributed; therefore, the hereditary form was suspected.

After admission, an echocardiogram showed an ejection fraction of 50% and abnormal wall motion with mild aortic and mitral valve regurgitation. His hemoglobin concentration was 11.3 g/dL, platelet count was 170,000/mm^3^, prothrombin time-international normalized ratio (PT-INR) was 2.54, and activated partial thromboplastin time (aPTT) was 43.6 s (control, 30 s). Warfarin was stopped 7 days before surgery, and the patient was started on intravenous unfractionated heparin to maintain an aPTT of 45 to 75 s (1.5–2.5 times control). Heparin was stopped 5 h before surgery. After anesthetic induction, the activated clotting time (ACT) was 142 s.

The patient received 720 mL of FFP prior to heparinization. Its volume was determined by taking the patient’s urine output into consideration to avoid heart congestion. Before the initiation of CPB, 300 units/kg of heparin was administered and the ACT was 536 s. The left and right internal thoracic arteries were grafted to the left posterolateral branch and the left anterior descending artery, respectively. The total CPB time was 138 min with an aortic cross-clamp time of 102 min, and the patient was weaned from bypass without complications. After a protamine dose of 3 mg/kg, the ACT was 126 s, while the bilateral internal thoracic artery graft flow was maintained at > 50 mL/min. An additional 1200 mL of FFP was transfused after protamine administration until the end of surgery. Its volume was decided, considering the CPB ultrafiltration volume. The amount of bleeding was 1200 mL and total intraoperative transfusion comprised 2240 mL of packed red blood cells, 1920 mL of FFP, and 200 mL of packed platelets.

In the intensive care unit, an additional 360 mL of FFP was administered. Nineteen hours postoperatively, the patient had no bleeding, and the heparin infusion was resumed with a dose adjustment to achieve an aPTT of 45 to 75 s. Warfarin was restarted 3 days after the surgery and adjusted to maintain a PT-INR of 1.5 to 2.5. The heparin infusion was discontinued after the PT-INR became stable. The postoperative period was uneventful, and the patient was discharged without any complications.

## Discussion

PS is a vitamin K-dependent protein produced in the liver and augments the effect of PC to inhibit activated coagulation factors V and VIII. Hence, PS acts as an anticoagulant factor [1]. The PS activity level is affected by hepatic or renal disorders, vitamin K antagonist (warfarin), chronic infection, pregnancy, disseminated intravascular coagulopathy, and other conditions, all of which results in acquired PS deficiency [4]. The incidence of PS deficiency in patients undergoing cardiac surgery reportedly ranges from 0.03 to 0.13% [5]. Because of this rarity, little evidence is available regarding avoidance of thromboembolism in patients with PS deficiency who undergo cardiac surgery.

Prophylactic anticoagulation therapy is recommended for PS deficiency patients exposed to surgery [2]. CPB may trigger consumptive coagulopathy because the blood is in contact with the surface of its circuit [6]. Thus, PS deficiency patients who undergo cardiac surgery involving CPB have a significantly increased risk of thrombosis. However, heparin must be discontinued intraoperatively to avoid hemorrhagic complications, necessitating other anticoagulants during the period of heparin cessation.

No purified form of PS is available for clinical use. FFP is the only agent that can enhance the level of PS [4]. Hsu and Despotis [3] described two patients with PS deficiency who underwent CABG, and FFP was transfused as the priming fluid of CPB. Balan [4] transfused FFP during the pre-bypass period and added more FFP for CPB priming. Neither of these reports focused on transfusing FFP during the period of heparin cessation. In these cases, FFP was not administered after protamine, increasing the potential risk of thrombosis. Our case is unique in that FFP was administered at pre-bypass and after protamine, while targeting two heparin cessation periods.

We did not measure the plasma PS level perioperatively because we assume that measurement of this parameter is problematic. First, it takes approximately 2 to 4 days to measure the PS level in our institution, making it difficult to adjust the volume of FFP while monitoring the PS level in real time intraoperatively. Second, approximately 60% of PS antigen is bound to C4b-binding protein (C4bBP) [1]. Acute inflammation triggered by CPB can result in an increase of C4bBP, which may lead to a reduction in free PS antigen by increasing its binding to C4bBP [1, 4]. In that case, a low level of free PS antigen does not necessarily indicate a shortage of FFP volume. Third, in our institution, the results of both PS activity and antigen are shown as a percent, whereas the cut-off value of the PS level shown as a percent for identification of the risk of thrombosis has not been fully elucidated. Lijfering and Pintao [7, 8] described the cut-off value of the free PS antigen level, but their data are presented in units of U/dL. The difference in units makes it difficult for us to refer to their data. Finally, Haubelt [9] described a wide variety of PS values contained in FFP, resulting in various increases in the plasma PS level after FFP infusion. Therefore, even if the cut-off value of PS is clarified, the volume of FFP needed to achieve an adequate PS level above the cut-off value would be difficult to predict.

The thrombin generation assay (TGA) (Technoclone GmbH, Vienna, Austria) evaluates procoagulant-induced thrombin generation and anticoagulant-induced thrombin decay [10]. The peak height, which represents the highest thrombin concentration, rises in a hypercoagulable state and falls in a hypocoagulable state. Heger [11] reported that the peak height decreased as the plasma PS level increased. Based on this finding, the TGA may be beneficial in that it can help to identify the real-time effect of FFP administration on PS level and clarify the most appropriate times and volume of FFP administration in PS deficiency patients. Unfortunately, application of the TGA is limited to laboratories in Japan. Although the clotting time, one of the parameters of rotational thromboelastometry (ROTEM; Pentapharm GmbH, Munich, Germany), had correlation with the PS level [11], its clinical usefulness for PS deficiency patients is not well known.

Patients with PS deficiency have historically received warfarin as an anticoagulant therapy [2]. However, warfarin decreases the PS level and potentially induces a hypercoagulable state, resulting in skin necrosis [12]. Xa inhibitors have been considered as potential alternative drugs [13]. Martinelli [14] described that skin necrosis disappeared after warfarin was altered to rivaroxaban. Little available evidence shows that Xa inhibitors are safer than warfarin with respect to skin necrosis [13].

Villacorta [15] reported a case of intraoperative graft thrombosis in a patient with PS deficiency after administration of protamine. The author recommended the use of a lower dose of protamine to avoid thrombosis. Our patient was administered 3 mg/kg of protamine, the typically administered dose, and no graft occlusion occurred. This outcome indicates that administration of FFP to augment the PS level may have a beneficial effect on avoiding graft thrombosis after protamine infusion.

## Conclusions

On-pump CABG was uneventfully performed in a patient with PS deficiency who underwent FFP transfusion targeting two intraoperative heparin cessation periods. FFP administration with consideration of the heparin cessation period may provide insight into the perioperative management of patients with PS deficiency undergoing cardiac surgery involving CPB, while some qualitative measurements such as TGA or ROTEM are desirable to evaluate the usefulness of our FFP administration strategy.

## Abbreviations

ACT: Activated clotting time
aPTT: Activated partial thromboplastin time
C4bBP: C4b-binding protein
CABG: Coronary artery bypass grafting
CPB: Cardiopulmonary bypass
FFP: Fresh frozen plasma
PC: Protein C
PS: Protein S
PT-INR: Prothrombin time-international normalized ratio
ROTEM: Rotational thromboelastometry
TGA: Thrombin generation assay
